# Identifying Quantitative Trait Loci for Thousand Grain Weight in Eggplant by Genome Re-Sequencing Analysis

**DOI:** 10.3389/fgene.2022.841198

**Published:** 2022-05-18

**Authors:** Zongwei Qian, Yanhai Ji, Ranhong Li, Sergio Lanteri, Haili Chen, Longfei Li, Zhiyang Jia, Yanling Cui

**Affiliations:** ^1^ National Engineering Research Center for Vegetables, Vegetable Research Institute, Beijing Academy of Agriculture and Forestry Sciences, Beijing, China; ^2^ Key Laboratory of Biology and Genetic Improvement of Horticultural Crops (North China), Ministry of Agriculture and Rural Affairs, Beijing, China; ^3^ Beijing Key Laboratory of Vegetable Germplasm Improvement, Beijing, China; ^4^ College of Life Sciences and Technology, Mudanjiang Normal University, Mudanjiang, China; ^5^ DISAFA, Plant Genetics and Breeding, University of Turin, Grugliasco, Italy; ^6^ Jingyan Yinong (Beijing) Seed Sci-Tech Co. Ltd., Beijing, China

**Keywords:** eggplant, thousand-grain weight (TGW), PG-ADI models, QTLs, ubiquitin-proteasome pathway

## Abstract

Eggplant (*Solanum melongena* L.; 2n = 24) is one of the most important Solanaceae vegetables and is primarily cultivated in China (approximately 42% of world production) and India (approximately 39%). Thousand-grain weight (TGW) is an important trait that affects eggplant breeding cost and variety promotion. This trait is controlled by quantitative trait loci (QTLs); however, no quantitative trait loci (QTL) has been reported for TGW in eggplant so far, and its potential genetic basis remain unclear. In this study, two eggplant lines, 17C01 (P1, wild resource, small seed) and 17C02 (P2, cultivar, large seed), were crossed to develop F1, F2 (308 lines), BC1P1 (44 lines), and BC1P2 (44 lines) populations for quantitative trait association analysis. The TGWs of P1, P2 and F1 were determined as 3.00, 3.98 and 3.77 g, respectively. The PG-ADI (polygene-controlled additive-dominance-epistasis) genetic model was identified as the optimal model for TGW and the polygene heritability value in the F2 generation was as high as 80.87%. A high-quality genetic linkage bin map was constructed with resequencing analysis. The map contained 3,918 recombination bins on 12 chromosomes, and the total length was 1,384.62 cM. A major QTL (named as TGW9.1) located on chromosome 9 was identified to be strongly associated with eggplant TGW, with a phenotypic variance explanation of 20.51%. A total of 45 annotated genes were identified in the genetic region of TGW9.1. Based on the annotation of Eggplant genome V3 and orthologous genes in *Arabidopsis thaliana*, one candidate gene SMEL_009g329850 (SmGTS1, encoding a putative ubiquitin ligase) contains 4 SNPs and 2 Indels consecutive intron mutations in the flank of the same exon in P1. SmGTS1 displayed significantly higher expression in P1 and was selected as a potential candidate gene controlling TGW in eggplant. The present results contribute to shed light on the genetic basis of the traits exploitable in future eggplant marker-assisted selection (MAS) breeding.

## Introduction

Eggplants (*Solanum melongena* L., 2n = 24) are highly present in our daily lives and are important members of the Solanaceae family. The total global cultivation of eggplants utilized 1,857,664 ha in 2019; among this land, China utilized approximately 782,998 ha, and India harvested 727,000 ha (FAOSTAT 2019; http://faostat3.fao.org). Eggplant seeds are important genetic propagules, and seed vigour, germination rate, colour and thousand-grain weight (TGW) are important targeted traits. TGW is especially important in eggplant seed production, as it affects eggplant breeding costs and the popularization of new varieties. There has been little research on the TGW of eggplant; in contrast, research on the genetic and molecular mechanisms of the TGW of other food crops has become increasingly important in plant science (Zuo and Li, 2014; Hu et al., 2019).

To date, abundant major QTLs and genes related to TGW in food crops that use seeds as products have been identified (Simmonds et al., 2014; Guan et al., 2018; Brinton and Uauy, 2019). The TGW of wheat is usually stably inherited (Kuchel et al., 2007), and it includes grain length, grain width, grain area and grain filling characteristics, which are also under independent genetic control (Gegas et al., 2010). Almost all wheat chromosomes have identified QTLs for grain weight (Huang et al., 2003; Gegas et al., 2010; Kumar et al., 2016; Zhang et al., 2016). In wheat, single loci often increase the average grain weight by <10%, and no genes have been cloned (Roder et al., 2008; Huang et al., 2015; Farre et al., 2016). Gene editing and mutation analysis have shown that TaGW2 negatively regulates the grain size and weight of wheat (Yang et al., 2012; Simmonds et al., 2014; Wang et al., 2018; Yi et al., 2018). Several studies on wheat have also revealed that certain genes, including TaTGW6, TaGW7, TaGW8 and TaGS-D1, are associated with grain weight (Zhang et al., 2014; Hu et al., 2016; Wang et al., 2019; Yan et al., 2019). Compared to the TGW QTL identified in wheat, the QTL of major grain weight in rice often increases grain weight by >20% (J. and C., 2019). In rice, TGW is an important determinant of yield, which correlates with grain plumpness, grain number per panicle and grain size (Chen et al., 2021). Many TGW QTLs of rice have been mapped to rice chromosomes (Guo et al., 2009; Liu et al., 2010; Bai et al., 2011; Tang et al., 2013; Xu et al., 2015; Zhang et al., 2016). Based on these or similar studies, many TGW-associated genes of rice have been cloned, such as the RING-type E3 ubiquitin ligase OsGW2 (Song et al., 2007), the arginine-rich domain nuclear protein OsGW5 (Shomura et al., 2008), the SBP domain transcription factor OsGW8 (Wang et al., 2012), and the indole-3-acetic acid (IAA)-glucose hydrolase protein OsTGW6 (Ishimaru et al., 2013). To date, QTLs/genes associated with TGW have been mostly cloned from cultivated rice (Li et al., 2020). Compared with related research in rice, the QTLs and molecular cloning of genes associated with TGW are lacking for maize (Guo et al., 2011; Liu et al., 2014). The gln1-4 (encoding a glutamine synthetase) was the first gene demonstrated by mutation analysis to influence maize kernel size (Martin et al., 2006). Through an orthologous cloning method, ZmGS3 and ZmGW2 in maize were identified to be highly homologous with OsGS3 and OsGW2 in rice (Li et al., 2010a; Li et al., 2010b).

In contrast with other food crops, for eggplant, many linkage maps have been constructed, and many agronomic trait QTL analyses have been conducted in recent decades (Wei et al., 2020a; Barchi et al., 2021). These traits include fruit-related traits (Cericola et al., 2014; Ezio et al., 2015; Toppino et al., 2016; Wei et al., 2020b), plant morphology (Lorenzo et al., 2012; Ezio et al., 2015) and eggplant diseases (Miyatake et al., 2016; Barchi et al., 2018) but do not include seed traits, such as TGW.

In our previous study, we constructed a high-density bin-mark genetic linkage map with the F2 population by using gene re-sequencing (Qian et al., 2021). In this study, we conducted further studies using this F2 population. The population was derived from two inbred lines, 17c01 (P1) and 17c02 (P2); P1 was a cultivation eggplant, and P2 was wild, with notably different TGW. The objective was to identify QTLs related to TGW, develop SNP markers and promote applications in eggplant molecular breeding for high seed yield.

## Materials and Methods

### Plant Materials

The female parent (P1) was the wild eggplant “17C01” (*Solanum insanum* (L.) *Banfi, Galasso & Bartolucci*), and the male parent (P1) was the cultivated eggplant “17C02” (*S. melongena* L.). The seeds were stored and supplied by the Eggplant Research Group of the Beijing Vegetable Research Center. “17C01” is a wild resource with many prickles, small round fruit (the average length is 2.20 cm, and the diameter is 2.80 cm), and small seeds, whereas “17C02” is an inbred line with few prickles, long rod fruit (the average length is 18.62 cm, and the diameter is 2.93 cm), and larger seeds. The P1, P2, F1, F2, BC1P1 and BC1P2 populations were used for the genetic analysis. The F1 population was generated from a cross between “17C01” and “17C02,” the F2 populations (308 seedlings) were generated from self-fertilization of F1, and the BC1P1 and BC1P2 populations, all containing 44 seedlings, were generated from F1 progeny backcrossed to P1 and P2, respectively. In the spring of 2018, all populations were grown in greenhouses at the experimental field of the Beijing Vegetable Research Center, Beijing Academy of Agriculture and Forestry, Beijing, China, with 120 cm between rows and 50 cm between each plant. The eggplant plants were grown in a standard manner until the fruits totally matured, and the fruits of each seedling were harvested. One thousand seeds were randomly selected and weighed.

Fresh eggplant leaves from the F2 mapping population and two parent populations were selected, and total genomic DNA was extracted by the CTAB procedure (Chen and Ronald, 1999).

### Phenotyping

The six eggplant populations P1, P2, F1 (P1 × P2), F2 (*n* = 308), BC1P1 (*n* = 44) and BC1P2 (*n* = 44) were tested via genetic analysis for TGW. After sowing, each eggplant seedling was individually numbered and shelfed separately, and the fruit were obtained 60 days after pollination. The seeds were harvested after 7 days of fruit post-maturation at room temperature. The TGW was measured by electronic scales (Sartorius BSA423S, Sartorius Stedim Biotech GmbH). Each phenotyping process was repeated three times, and the average value was utilized for further analysis. Phenotypic data analysis was performed using IBM SPSS v19.0 (SPSS Inc., IBM Corp., Chicago, IL, United States). The significance level was 0.05.

### Statistical Analysis of Inheritance

Six generations were used to perform quantitative genetic analysis of the TGW of eggplants. Following the segregation analysis of the genetic system of Gai (2006), the R Language software package SEA was used to analyse the genetic models of plant quantitative traits. Akaike’s information criterion (AIC) and a set of tests, including the uniformity test, Smirnov test, and Kolmogorov test (*U*_*1*_^*2*^ *, U*_*2*_^*2*^ *, U*_*3*_^*2*^ *,*_*n*_ *W* ^*2*^ *, D*_*n*_), were used to choose an optimal genetic model from the 24 genetic models. The 24 genetic models contained five types: the one-major-gene model, two-major-gene model, pure polygene model, one-major-gene plus polygene model, and two major-gene plus polygene model, and each type considered the additive, dominance, and epistasis effect of the major gene and polygene in various segregating generations. By using the method of least squares according to the relationship between the genetic parameters and the component distribution parameters of a given model, the estimates of genetic parameters, the additive, dominance, and epistasis effects of major gene(s), the total additive, dominance, and epistasis effects, the heritability of major genes, and the heritability of polygenes of the optimal genetic mode were calculated (Gai, 2006). The inheritance of the TGW of eggplants was calculated by using those methods.

### QTL Analysis Using a High-Density Genetic Linkage Map

The research team constructed a genetic linkage map by using the two parents and 100 randomly selected F2 individuals (Qian et al., 2021). Biomarker Technology Co., Ltd. (http://www.biomarker.com.cn/) completed the resequencing. DNA sequencing was performed on an Illumina HiSeq2500 (Illumina, Inc., San Diego, CA, United States). The clean reads were aligned to the S. melongena reference genome (i.e., Eggplant genome consortium V3) (https://solgenomics.net/organism/Solanum_melongena/genome) assembly by using Burrows–Wheeler Aligner (BWA) software (Li et al., 2009). One bin mark contains one or more SNPs. Bin marker delineation was performed based on offspring recombination. The bin marker screening rules were as follows. 1) Bin marks with lengths less than 10 kb were filtered. 2) The polymorphic bin markers with severe bias separation (*p* < 0.001 by chi-square test) were filtered.

QTLs related to TGW were identified by using a high-density genetic linkage map. QTLs with a logarithm of odds (LOD) value of 3.00 based on 3,000 replications at α = 0.05 and a walk speed of 1.0 cM were detected by using the forward and backward regression method. The intervals with two supported LODs were established as 95% confidence intervals (van Ooijen, 1992). Loci with the maximum LOD score were chosen as putative QTLs for the TGW of eggplant, and the names were based on the trait abbreviation (TGW) followed by a chromosome number. The values of the phenotypic variance rate, contribution rate and additive effects of the QTLs were calculated using the software.

### Analysis of Candidate Genes Regulating TGW

Functional annotation of candidate genes was compared with the Eggplant Genome Consortium V3 (https://solgenomics.net/organism/Solanum_melongena/genome) (Barchi et al., 2019), and *Arabidopsis* orthologous gene information of candidate genes was obtained with TAIR (https://www.arabidopsis.org) and candidate genes were further analysed, including annotation of the biological process and molecular function.

### Candidate Gene Expression Analysis

Total RNA was obtained from the seeds of “17C01” and “17C02” using an RNeasy kit (Qingke, Beijing, China). The seed coat was destroyed by using liquid nitrogen and then ground in liquid nitrogen. RNA was converted into cDNA using a Fast Quant RT Kit (Qingke, Beijing, China). qRT–PCR was performed in a 20-μl total volume using SYBR Green I Master Mix (Qingke, Beijing, China). The EF1α gene was used as an internal control, and all the primers are listed in Supplementary Table S1.

The protein sequences of SmGTS1 were downloaded (https://solgenomics.net/organism/Solanum_melongena/genome) and BLAST searched in NCBI (https://www.ncbi.nlm.nih.gov/genome). The top 15 protein sequences with the closest homology relationship were screened out (Expect = 0.00, Identities>87.00%), and AtGTS1 in Arabidopsis was added for phylogenetic analysis. The cluster W method embedded in MEGA 6.0 software was used for sequence alignment, The phylogenetic analysis was inferred by using the Maximum Likelihood (ML) method with MEGA6 software (Tamura et al., 2013).

## Results

### Phenotypic Characterization of the Eggplant TGW

**FIGURE 1:**
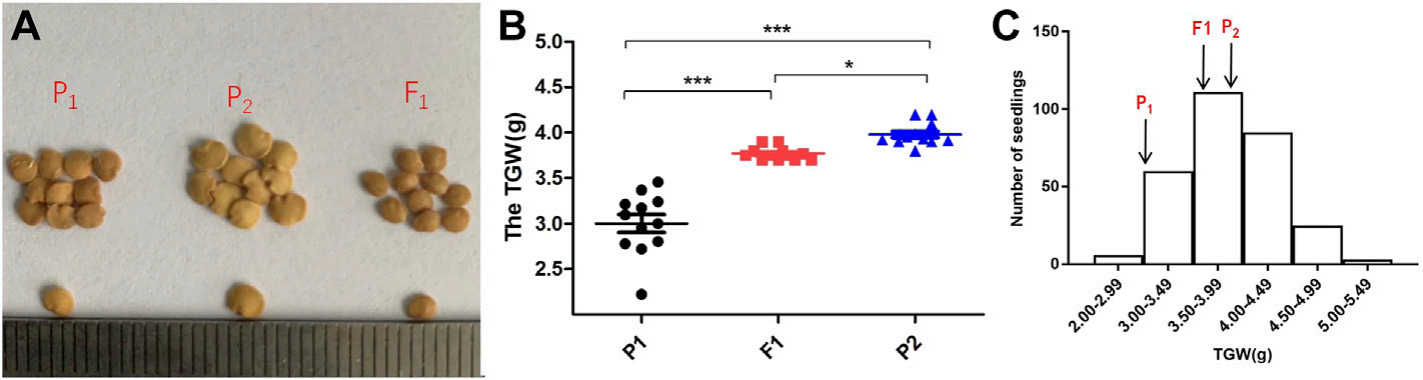
The phenotypic in parents, F1and segregating populations. **(A)**: Performance of the size of the parents (17c01 and 17c02) and the F1. **(B)**: The differences distribution of average TGW of P1, P2 and F1 population. **(C)**: Frequency distribution of the TGW in the F2 population. Arrows indicate the TGW of P1, P2 and the F1 population.

To investigate the QTL controlling the development of TGW in eggplant, the segregating populations were obtained by crossing “17C01” and “17C02.” 17C01, a wild landrace with small seeds, was used as the female parent (P1), while 17C02, an inbred line with larger seeds, was used as the male parent (P2). The size of F1 seeds was intermediate to the parent seed sizes (Figure 1A). The TGW values in the P1, P2, F1, BC1P1, BC1P2 and F2 populations were distinct (Supplementary Table S2). The two backcross populations BC1P1 and BC1P2 were obtained by crossing F1 to P1 and P2, respectively. The seedlings did not harvest seeds marked N/A, so the valid data of BC1P1, BC1P2 and F2 were 41, 42 and 290 seedlings, respectively. The TGW of the segregating populations was examined (Figure 1B). The average TGW of P1, P2 and F1 were 3.00, 3.98 and 3.77 g, respectively. The TGW of P1 was significantly different from that of P2 and F1, and F1 seeds were intermediate to the parents and closer to P2. The distribution of TGW in the F2 population was normal, indicating that the TGW of eggplants was a quantitative trait and suitable for QTL identification (Figure 1C and Supplementary Table S2).

### Inheritance of TGW in Eggplant Populations

To clarify the inheritance of the TGW of eggplant, the six generations P1, P2, F1, BC1P1 (B1), BC1P2 (B2), and F2 were used for quantitative genetic analysis. The values of the log maximum likelihood functions and Akaike’s information criterion (AIC) of 24 genetic models were calculated through the IECM algorithm. The MX2-A-AD models had the lowest AIC value, and the AIC values of PG-ADI and MX1-AD-ADI were near those of MX2-A-AD. Therefore, the three models were selected for the candidate models (Supplementary Table S3). Tests for the quality-of-fit of the three candidate models (MX2-A-AD, PG-ADI and MX1-AD-ADI) showed that the AIC value of the MX1-AD-ADI model was lowest, but this model contained three values with a significant difference, so the MX1-AD-ADI model was not the optimal genetic model. Tests for the quality-of-fit of the other two models, PG-ADI and MX1-AD-ADI, all had no significant difference values. The PG-ADI models had lower AIC values than those of the MX1-AD-ADI model, so the PG-ADI model (polygene-controlled additive-dominance-epistasis genetic model) was the optimal inheritance model for the TGW of eggplant (Supplementary Table S4).

The least square method was used to calculate the values of the first-order and second-order genetic parameters and the effect values and heritability of the major gene or polygene in the optimal genetic model (Table 1). Because the PG-ADI models were the optimal inheritance model, there were no major genes controlling the TGW of eggplant. According to the heritability results, the polygene heritability values of the B1, B2 and F2 generations were 83.65, 70.11 and 80.87%, respectively.

**TABLE 1:** Estimates of genetic parameter of the TGW of eggplant.

1st Order parameter	Estimate	2nd Order parameter	Estimate		
			B_1_	B_2_	F_2_
m	3.00	σ^2^_p_	0.28	0.15	0.24
		σ^2^_pg_	0.23	0.11	0.19
		h^2^_pg_ (%)	83.65	70.11	80.87

Note: m: Average of the population; σ^2^_p_: Phenotypic variance; σ^2^_pg_: Multi-gene variance; h^2^_pg_: Polygene heritability.

These results indicated that the TGW of eggplant was inherited through polygenes and had high heritability in the offspring.

### QTL Mapping for the TGW Trait

The Illumina HiSeq platform was used to re-sequence the whole genome of the two parents and 100 randomly F2 individuals, and the data and the map have been published in previous research papers (Qian et al., 2021). A total of 278,891 bin markers containing 1,883,288 SNPs were obtained on 12 chromosomes. The high-density bin genetic map contained a total of 3,918 recombinant bins, and the total length was 1,384.62 Cm.

**FIGURE 2:**
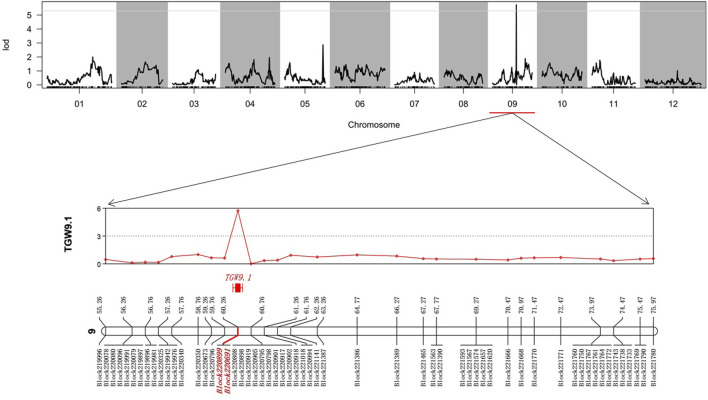
Quantitative trait locus (QTL) regions associated with the TGW. The markers within QTL regions associated were highlighted with red.

The values of LOD, additive, dominance and phenotypic variation explained (PVE) for the QTLs of TGW in the whole genome are listed (Supplementary Table S5). As a result, we identified one significant QTL, TGW9.1; the physical distance was 26,099,638-26,772,016 (67,378 bp), and the genetic position was 60.26 cM on chromosome 09 (Figure 2). This QTL harboured two bin marks, Block 220899 (26751908-26771916) and Block220691 (26099638-26110816), and the LOD values were both high at 5.73.

A QTL with PVE lower than 15% was defined as a minor QTL; otherwise, it was defined as a major QTL (Mahasuk et al., 2016). The PVE of the QTL of the TGW of eggplant (TGW9.1) was 20.51% (Table 2). These results suggested that the TGW trait of eggplant was regulated primarily by one major QTL (TGW9.1) with two bin marks on chromosome 9, which we considered to be the candidate genomic regions. In addition, the additive and dominant effects of TGW9.1 were negative, indicating that parent 17c02 contributed favourable alleles for TGW.

**TABLE 2:** The QTL TGW9.1 identified for the TGW of eggplant.

QTL	Chr	Physical distance interval (bp)	Genetic position (cM)	Bin mark number	LOD	PVE%	Additive	Dominant
TGW9.1	9	26099638–2,6772016	60.26	2	5.73	20.51	−0.25	−0.18

Note: LOD: logarithm of the odds; PVE: The phenotypic variation explained by the QTL (%); Additive, Dominant: the effect with positive and negative value indicating contribution of P1 and P2, respectively.

### Candidate Gene Analysis for the Major QTL TGW9.1

There were 45 genes in the candidate genomic regions on 60.26 cM chromosome 9; among these genes, 23, 22, 38 and 45 genes had annotation information in the GO, KEGG, Swiss-Prot and nr databases, respectively (Supplementary Table S6).

Many studies have shown that plant seed size is controlled by some key genes and regulatory pathways, such as the ubiquitin–proteasome pathway, G protein signalling, some transcriptional regulators, mitogen-activated protein kinase (MAPK) signalling, phytohormone perception and homeostasis (Li and Li, 2016; Hu et al., 2019; Wang et al., 2021). According to the annotation information, there were only three genes (SMEL_009g329890, SMEL_009g329850, SMEL_009g329560) related to the ubiquitin–proteasome pathway, and the other genes were not related to the above pathways, so we considered the three genes to be potential candidate genes of the TGW of eggplant. One candidate gene is SMEL_009g329890, and the GO annotation information showed that it has molecular functions related to ubiquitin-protein transferase activity (GO: 0004842). The homologous gene of SMEL_009g329890 in Arabidopsis is BAH1/NLA (AT1G02860), which encodes a ubiquitin E3 ligase with RING and SPX domains. E3 ubiquitin ligases have been shown to play major roles in cell growth and stress response in Arabidopsis (Xu et al., 2017). The second candidate gene was SMEL_009g329850, for which the GO annotation information is the Cellular Component: Cul4-RING E3 ubiquitin ligase complex (GO: 0080008). The homologous gene of SMEL_009g329850 in Arabidopsis is AtGTS1 (AT2G47790), which is a transducin/WD40 protein superfamily member and controls seed biomass accumulation, growth and germination (Gachomo et al., 2014). The third candidate gene was SMEL_009g329560, which affected the biological process of ubiquitin-dependent protein catabolic process (GO: 0006511). The homologous gene of SMEL_009g9560 in Arabidopsis is CUL1 (AT4G02570.1), which encodes a cullin that is a component of SCF ubiquitin ligase complexes involved in mediating responses to auxin and jasmonic acid in Arabidopsis (Quint et al., 2005) (Table 3).

**TABLE 3:** The candidate genes of the TGW of eggplant.

QTL	Gene ID	Arabidopsis homolog			
		Name	Type	Symbols	Annotation
TGW9.1	SMEL_009g329890	AT1G02860	protein coding	BAH1/NLA	Encodes a ubiquitin E3 ligase
	SMEL_009g329850	AT2G47790	protein coding	GTS1	Encodes GIGANTUS1 (GTS1), a member of Transducin/WD40 protein superfamily
	SMEL_009g329560	AT4G02570	protein coding	CUL1	Encodes a cullin

**FIGURE 3:**
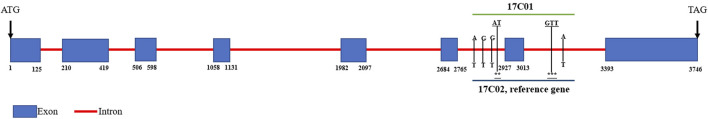
The genomic sequence alignments of SMEL_009g329850 between 17C01 and 17C02. The black rectangles and red lines were designated as exons and introns, respectively. The vertical bar, 17C01 and 17C02 were placed on the up and down, respectively, represent nucleotide variation and the deletion, the * represented that there was no nucleotide in this site.

Therefore, the sequence differences of these three genes between 17C01 and 17C02 were examined (Figure 3 and Supplementary Figure S1). The results showed that there were 2 single nucleotide variations (all in the intron region) in SEML_009g329890 between 17C01 and 17C02. In SMEL_009g329560, there was only one single nucleotide variation in the intron region between the parents. In SMEL_009g329850, there were a total of 4 single nucleotide variations and 2 indels (3 bp and 2 bp) between P1 (7C01) between P1 (7C01) and P2 (17C02). All the variant sites were in the intron region, but all were located on both sides of the same exon. These deletions may cause alterations in its function, resulting in a difference in the TGW of the parents. These results suggest that SMEL_009g329850, named SmGTS1, might be a key candidate gene related to the TGW of eggplant.

**FIGURE 4:**
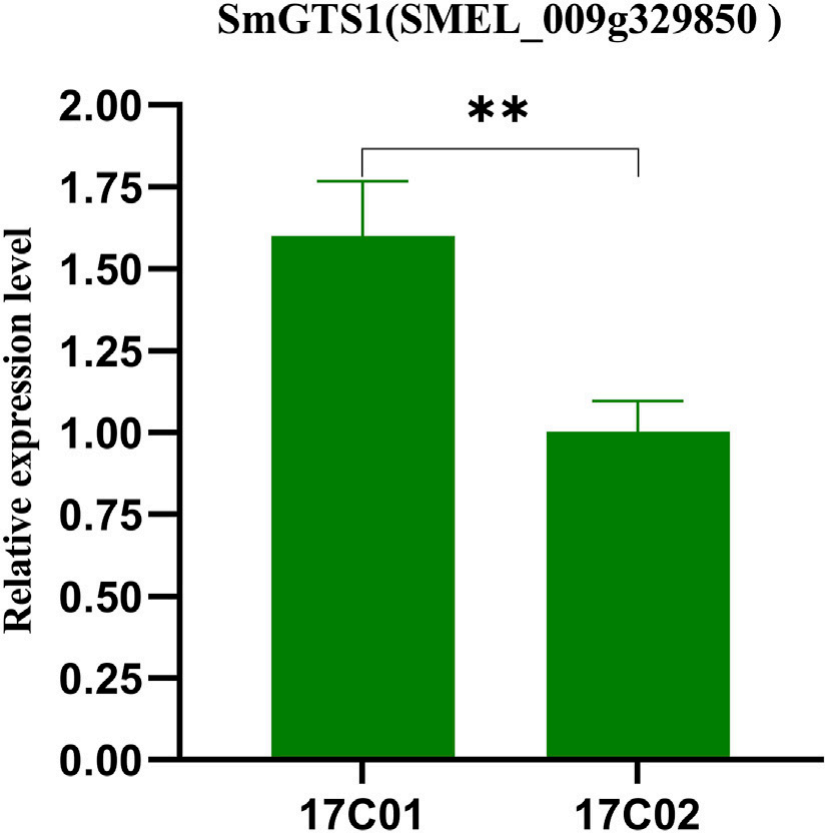
Gene expression levels of the SmGTS1 in the parents (17c01 and 17c02). Expression of SmGTS1 in the seeds of the 17c01 and 17c02. Values are the mean ± SD of three biological replicates. **: significantly different at *p* < 0.01.

The expression levels of the key candidate genes in the seeds of 17C01 and 17C02 were examined by using qRT–PCR. The expression of SmGTS1 in P1 was significantly higher than that in P2 (*p* = 0.0057), and the higher expression level of SmGTS1 in eggplant probably conferred smaller seed sizes (Figure 4). These findings suggest that SmGTS1 is the most likely key candidate gene for the regulation of TGW in eggplant.

**FIGURE 5:**
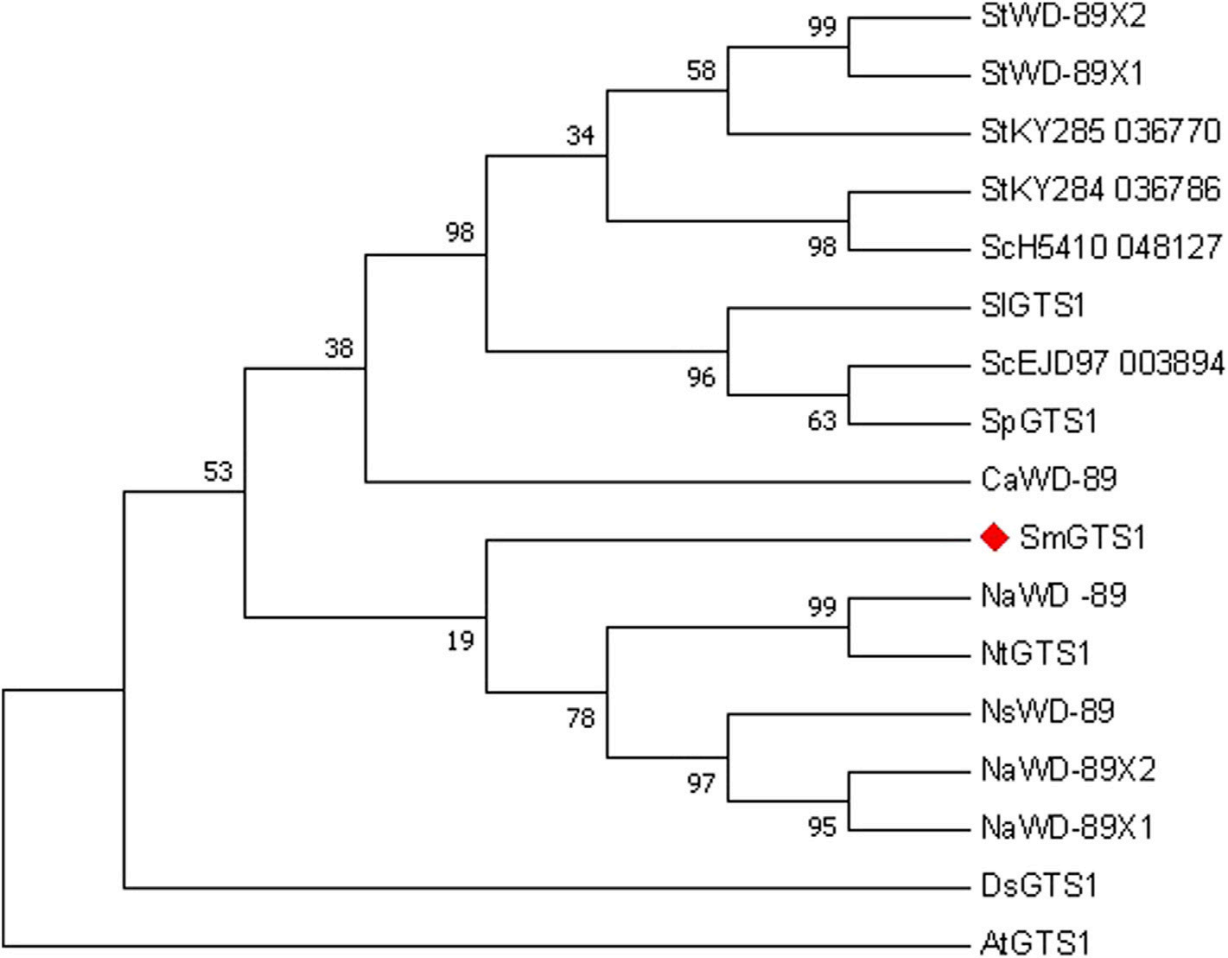
Phylogenetic analysis of SmGTS1 by Maximum Likelihood method. The percentage of replicate trees in which the associated taxa clustered together in the bootstrap test (1,000 bootstrap replications) are shown next to the branches. Protein name: the initials of the species name, and the protein features. The SmGTS1 was marked with a red diamond.

The GTS protein is a WD40 repeat-like superfamily protein member. The protein sequences of SmGTS1 and its homologous genes in potato, tomato, pepper, tobacco, Solanum nigrum, mandala and Arabidopsis were used for phylogenetic analysis (Figure 5 and Supplementary Table S7). The results showed that the SmGTS1 protein in eggplant had higher homology with WD or GTS1 protein in the same Solanaceae plant, such as tobacco, potato, tomato and pepper, and had lower homology with AtGTS1 in Arabidopsis.

## Discussion

Plant seeds are important organs in which genetic material is transmitted to the next generation. For wheat, rice and maize, in which seeds are the main product organ, seed size and weight are very important agronomic traits (Guo et al., 2011; Wang et al., 2019; Miao et al., 2020). The TGW of plants can influence plant fitness, adaptation to environmental stresses, yield and quality. Therefore, the TGW of food crops was the key characteristic examined in the study. With vegetables, there have also been some studies on the TGW of dual-use seeds of Cucurbitaceae vegetables, such as pumpkin and watermelon (Guo et al., 2020; Wang et al., 2021). Eggplant and other Solanaceae vegetables use fruit as the edible organs, so researchers pay less attention to the TGW of eggplant, and the underlying molecular mechanisms remain unclear. However, TGW is very significant for the commercial breeding of eggplant, and a larger TGW and high seed yield are important in breeding, which means that the cost is low and the efficiency is high in parental lines and hybrid reproduction. In this study, the TGW of eggplant was used as the research objective, the QTL for TGW was mapped, and three candidate genes were obtained. This research provides a theoretical basis for eggplant breeders and commercial breeding companies.

The combination and interaction between quantitative genetics and plant breeding have promoted very significant progress in cultivar development (Gai, 2006). According to the genetic model results, the PG-ADI model was the optimal genetic model for the TGW of eggplant, which showed that the TGW of eggplant was inherited mainly via inheritance of the polygene based on the additive-dominance-epistasis fit. Through genetic parameter analysis, the polygene heritability of the F2 generation was as high as 80.87%. These results show that there were more than two genes controlling the TGW of eggplant in one or more chromosomes and that those genes had high heritability. Therefore, there may be other related QTLs on other chromosomes according to the genetic model, but in this study, we found only one major QTL (PVE = 20.51%) on 9 chromosomes. The above possibilities suggest that there are almost no other relevant studies on the TGW of eggplant for reference. In future research, we may change the genetic mapping materials to find other QTLs and verify this QTL.

In this experiment, we used the V3 version of the eggplant genome as the reference genome. In the same year the experiment ended, the same research team published a novel, highly contiguous genome version based on Hi-C (Barchi et al., 2019; Barchi et al., 2021). We blast searched the key candidate gene CDS using version 4 (Supplementary Table S8). We found that the CDS of the candidate gene was the same between the v3 and v4 versions (id% = 100, evalue = 0.0), and they were named SMEL_009g329850 and SMEL4_09g007390, respectively. All were located on chromosome 9, and the description showed that they were similar to the GTS1 WD repeat-containing protein GTS1. The above results show that SMEL_009g329850 and SMEL4_09g007390 were the same genes in different genomes and were related to GTS1 WD repeat-containing proteins. In addition, this experiment using the V3 version of the eggplant genome was reliable and suitable for this experiment. In addition, the results of this experiment may demonstrate that the V3 and V4 versions of eggplant genomes have good continuity and reliability.

GIGANTUS1 (GTS1) regulates growth and development in plants and is a member of the transducin/WD40 protein superfamily (Gachomo et al., 2014). Transducin/WD40 repeats are prominent features within proteins that mediate diverse protein–protein interactions, including those involved in scaffolding and the cooperative assembly and regulation of dynamic multisubunit complexes (Gibson, 2009). WD40 domains were first described in bovine β-transducin, which has 44–60 residue sequence repeats as characteristic sequence and structural features (Fong et al., 1986; Stirnimann et al., 2010). These domains are more abundant in eukaryotic organisms and play important roles in biological processes, such as light signalling and vision, apoptosis, cell division and cytokinesis, cell motility, meristem organization, cytoskeleton dynamics, protein trafficking, chemotaxis, nuclear export to RNA processing, chromatin modification, transcriptional mechanisms, flowering and floral development (Stirnimann et al., 2010). In Arabidopsis, AtGTS1 regulates seed germination and growth development (biomass yield and flowering time) (Gachomo et al., 2014). In this study, the key candidate gene was SmGTS1, and the homologous gene in Arabidopsis was AtGTS1. According to the functional analysis of Arabidopsis, the TGW of eggplant may be influenced by SmGTS1. Real-time PCR of SmGTS1 in parents showed that the SmGTS1 gene may negatively regulate seed weight. We also found related homologous genes and proteins in Nicotiana and Solanum family plants, but lack functional analysis of those related genes.

Introns are found across almost all species, and alternative splicing of introns can lead to an expansion of the protein repertoire of organisms, increase complexity and phenotypic diversity (Bush et al., 2017). Many studies have shown that many introns are able to enhance the expression of their respective genes by a significant amount (Clancy and Hannah, 2002; David-Assael et al., 2006). In this study, there were consecutive intron mutations (4 SNPs and 2 Indels) in the flank of the same exon (2,927 bp-3013 bp) of the SmGTS1 gene in 17C01. This may lead to alternative splicing of the SmGTS1 gene, which may alter gene expression and change the TGW of eggplant. The results of Real-time PCR of SmGTS1 in parents also confirmed these findings.

Many studies have shown that seed formation and development is a complex process. Several ubiquitin-related activity factors have been known to influence seed size. The ubiquitin receptor DA1 is a growth-restricting factor that contains two ubiquitin interaction motifs (UIMs) and can bind ubiquitin *in vitro*, and the da1-1 mutant forms in the Arabidopsis maternal integuments of ovules can increase seed size (Li et al., 2008). In rice, a QTL for GRAIN WIDTH AND WEIGHT2 (OsGW2), encoding a previously unknown RING-type E3 ubiquitin ligase, regulates grain size by restricting cell division (Song et al., 2007). Together with BB/EOD1 and the E3 ubiquitin ligase DA2, the ubiquitin receptor DA1 can regulate seed growth in Arabidopsis (Xia et al., 2013). There were some other key genes and regulatory pathways, including mitogen-activated protein kinase (MAPK) signalling, G protein signalling, phytohormone perception and homeostasis, and some transcriptional regulators that can control seed size and weight. In this study, the key candidate gene was related to the ubiquitin–proteasome pathway. The results showed that the ubiquitin–proteasome pathway may be an important regulatory pathway in eggplant seed size regulation. The key candidate gene prediction provides a reference for analysing the genetic basis of TGW in eggplant and breeding based on this trait.

## Conclusion

In this study, we first performed quantitative genetic analysis and defined an optimal inheritance model of TGW. Additionally, we constructed a high-quality genetic linkage map by whole genome resequencing of 100 F2 individuals. The major QTLs conferring TGW were identified. The ubiquitin–proteasome-related gene SmGTS1 in QTLs (TGW9.1) on chromosome 9 was proposed as a key candidate gene controlling the TGW of eggplant. The inheritance model, map, QTLs and key candidate genes obtained by this study will provide references for future basic and applied research involving TGW traits in eggplant.

## Data Availability

The datasets presented in this study can be found in online repositories. The names of the repository/repositories and accession number(s) can be found below: https://ngdc.cncb.ac.cn/gsa, CRA004017.
