# Supplementary figures and images for: Identifying Quantitative Trait Loci for Thousand Grain Weight in Eggplant by Genome Re-Sequencing Analysis
Source: Front Genet. 2022 May 18;13:841198. doi: 10.3389/fgene.2022.841198 (PMC9157640; doi:10.3389/fgene.2022.841198)

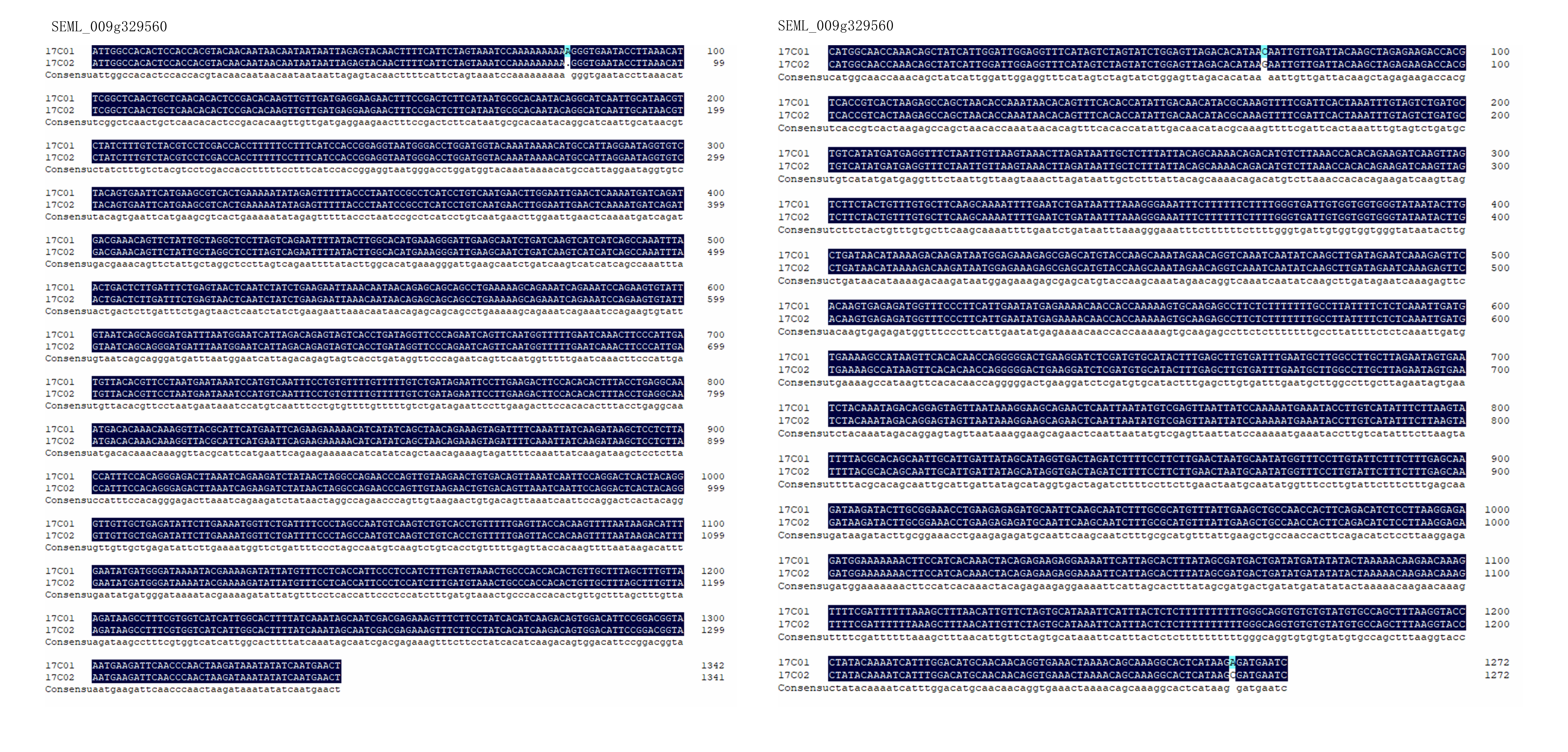

Supplement: Supplementary file 2 [file Image1.JPEG]
